# Effect of potassium fulvate on continuous tobacco cropping soils and crop growth

**DOI:** 10.3389/fpls.2024.1457793

**Published:** 2024-09-19

**Authors:** Yingle Jiao, Qian Chen, Xiaomeng Guo, Hongliang Li, Xuwei Chen, Kuifu Men, Xiaochen Liu, Xianchao Shang, Yun Gao, Li Zhang, Long Yang, Xin Hou

**Affiliations:** ^1^ College of Plant Protection, Shandong Agricultural University, Tai’an, China; ^2^ Shandong Nongda Fertiliser Sci. & Tech. Co. Ltd., Tai’an, China

**Keywords:** tobacco, potassium fulvate fertiliser, rhizosphere microorganisms, soil improvement, diseases

## Abstract

Long-term continuous cropping of tobacco causes dysbiosis of soil microbial communities, the imbalance of soil nutrients, and the increase of pathogenic bacteria, which will slow the growth and development of tobacco plants, reduce the production quality, and cause significant losses to tobacco production and tobacco farmers. The application of Potassium fulvic acid can not only provide nutrients, but also inhibit the propagation of pathogens in soil along with raising the amount of organic matter in the soil, which is an effective way to improve soil health. In this experiment, Tobacco variety SNT60 was used as the test material, and 6 treatments were set up by pot test, they were: no fertilisation control group (CK), tobacco special fertiliser (NPK), 3.45 g/kg of potassium fulvic acid fertiliser (T1), 4.65 g/kg of potassium fulvic acid fertiliser (T2), 5.85 g/kg of potassium fulvic acid fertiliser (T3), 7.05 g/kg of potassium fulvic acid fertiliser (T4), Ten replications were set up for each treatment and the soil and fertiliser were mixed and potted before transplanting, 70% as basal fertiliser and 30% as supplementary fertiliser. We also analyzed soil properties, soil microorganisms and agronomic traits of tobacco plants in different treatments to provide reference for mitigating tobacco succession barrier. The test results are as follows: 4.65 g/kg of potassium fulvic acid fertiliser (T2) treatment was the best, soil organic matter, quick nitrogen, phosphorus, potassium, pH, soil catalase, soil sucrase, and soil urease content, compared to CK control, increased by 22.04%, 43.12%, 96.21%, 381.79%, 25.43%, 91.69%, 262.07% and 93.16%. In terms of microbial community, application of potassium fulvic acid fertiliser significantly increased the relative abundance of Ascomycetes, Chlorobacterium, Bacillus, Proteobacteria and Tephritobacterium in the soil. Meanwhile, 4.65 g/kg of potassium fulvic acid fertiliser (T2) promoted the growth of tobacco plants, improved leaf photosynthetic capacity, and enhanced plant disease resistance. This experiment provides practical measures to improve the microbial community of tobacco continuous cropping soils and to reduce the incidence of diseases.

## 1 Introduction

Tobacco (*Nicotiana tabacum* L.) is an important cash crop in China, its planting area and production ranked first in the world, to promote the development of China’s national economy, to meet the people’s needs for a better life and so on (Chen et al., 2022a). According to statistics, in 2021, the tobacco industry achieved a total industrial and commercial tax and profit of 1358.1 billion yuan, an increase of 6.08 per cent year-on-year, with a total fiscal amount of 1244.2 billion yuan, making a positive contribution to economic development. As China’s population continues to grow, land resources are becoming more and more constrained, while the increasing degree of crop intensification has contributed to the increasing prevalence of continuous cropping (Chang et al., 2022). Growing the same crop continuously or in multiple cropping on a single piece of land is known as continuous cropping (Nazir et al., 2024). Prolonged mono-cropping creates specific soil microenvironments that increase the chances of pathogenic microbes in the soil, which can easily lead to a decline in crop yields. Chronic nutrient imbalances, on the other hand, can lead to shortages of certain essential elements in the soil and inhibit crop growth (Chen et al., 2022b). Continuous cropping leads to a decline in soil fertility and promotes a yearly increase in pathogenic bacteria and other organisms in the soil, which can lead to the development of soil-borne diseases (Chen et al., 2022b). Tobacco belongs to the tobacco genus of the Solanaceae family and is a crop in which mono cropping should be avoided, economic losses due to continuous cropping are as high as $4 billion per year. It has been found that the long-term use of organic fertilisers promotes soil microbial biomass activity and diversity, which in turn promotes crop growth and increased yields (Chen et al., 2018).

The rhizosphere harbours a large number of soil microorganisms and tiny soil fauna and is considered one of the most active regions in the world. These microorganisms play an important role in nutrient cycling, although they make up a small proportion of the soil. Moreover, plants and microorganisms are mutually beneficial, with plants providing nutrients to soil microorganisms through dead leaves and secretions, and microbial communities enhancing nutrient uptake by plants and combating plant pathogens. Fungi, as soil microorganisms, are also one of the most important indicators for assessing soil fertility and health. Fungi have a wide range of growth and resistance, and can directly participate in soil ecological processes such as soil organic matter cycling and nutrient transformation. In addition, fungi have a stronger ability to degrade organic matter than bacteria, and can also form mycorrhizae and crop symbiosis. Long-term use of chemical fertilisers reduces the diversity of fungal communities, and studies have shown that the use of organic fertilisers can change the composition and abundance of fungal communities, and reduce the number of disease-causing bacteria in the rhizosphere zone of crops (Wei et al., 2011).

Fulvate (Fulvic acid) is an important component of humic acid-like biomass that are more readily absorbed by plants. Fulvic acid has several sources in nature (Lang et al., 2020). Its main sources include a larger molecule of biochemical fulvic acid (BFA) formed by biofermentation of straw and by-products such as bio-ash (Brännvall et al., 2015) and formed from plant and animal residues (e.g. lignite, peat, etc.) through the transformation of soil microorganisms, and industrially extracted as a black, low molecular weight, mineral-derived fulvic acid (MFA) rich in hydroxyl, phenolic, carboxyl and other reactive functional groups, and soluble in acids, alkalis, and organic matter. It was found that spraying fulvic acid at moderate concentrations increased photosynthetic rate and water utilisation in maize, while IAA and ABA contents of maize leaves were also significantly higher (Anjum et al., 2011; Gao et al., 2022).

Potassium fulvic acid is a product made by reacting fulvic acid as raw material with potassium hydroxide or potassium carbonate, which can not only be applied alone, but also be used as foliar spraying or as a trailing fertiliser, and it can also be applied in combination with all kinds of chemical fertilisers to improve the usage rate of chemical fertilisers (Abdelrasheed et al., 2021). It has been found that the application of potassium fulvic acid can promote plant rooting and germination, improve the soil environment of crops, increase the absorption of nutrients by crops, and enhance the resilience of crops, etc (Abdelrasheed et al., 2021). It has been found that foliar spraying of Potassium fulvic acid promotes the uptake of nitrogen and potassium in tomato leaves, thereby increasing tomato yield (Xu et al., 2022). Potassium humate was found to promote ginger root growth and nutrient uptake, and to improve potassium utilisation. It has also been found in sweet potato that promoting crop growth improves potassium utilisation (Hassanpanah, 2011; Idrees et al., 2020). It was found that the application of potassium fulvic acid, can improve soil physico-chemical and also increase soil microbial communities (Pradip et al., 2016). It was found that the application of potassium fulvic acid mixed with lime improved the physico-chemical properties of the soil (Sadej et al., 2019). Studies have shown that potassium fulvic acid is effective in controlling pests and diseases (Jiali et al., 2015; Maji et al., 2017). Studies have shown that application of humic acid can increase the number of soil microorganisms (Li et al., 2022b). The results of a 3-year trial to study the improvement of soil properties and soil microbial diversity in continuous peanut cropping with humic acid showed that the same results were observed for three consecutive years found that humic acid improved the physico-chemical properties of the soil and that the soil microbial diversity was found to be highly variable according to high-throughput sequencing analysis, with a decrease in the number of bacteria and an increase in the number of fungi after humic acid treatment, which was beneficial in alleviating the peanut crop succession disorder (Li et al., 2019). The application of potassium fulvic acid can optimise the structure of microbial communities in the soil and intensify the competitive advantage of beneficial bacteria, thus improving the ecological environment of the soil and increasing soil fertility (Li et al., 2018).

In this study, the effects of potassium fulvic acid origin on the improvement of continuous cropping soil and the growth of tobacco were investigated through pot culture experiments. By analysing the physical and chemical properties of soil, microbial colonies and the growth and development of tobacco, the optimum amount of potassium fulvate to improve the continuous cropping soil of tobacco was investigated, with a view to providing a theoretical basis for the application of potassium fulvic acid on tobacco.

## 2 Materials and methods

### 2.1 Study site and test material

The experiment was set up in the greenhouse of the Plant Protection Experiment Station of Shandong Agricultural University (117°5′E and 36°43′N), and the soil chosen was a tobacco continuous cropping soils with a pH value of 6.06, an organic matter of 16.78 g/kg, an alkaline dissolved nitrogen (ADN) of 40.87 mg/kg, a quick-acting phosphorus (QAP) of 76.51 mg/kg, and a quick-acting potassium (K) of 75.56 mg/kg.

Varieties for testing: The pot test variety was Eggplant Coat SNT60, provided by the Department of Tobacco, College of Plant Protection, Shandong Agricultural University.

Fertilizers for testing: Potassium fulvic acid was provided by Shandong Nongda Fertilizer Science and Technology Co. Ltd, granular calcium superphosphate was provided by Hubei Fengle Ecological Fertilizer Co. Ltd, potassium sulphate was provided by Shandong Qingshang Chemical Co. Ltd, calcium ammonium nitrate for agricultural use was provided by Shenzhen Wugu Network Science and Technology Co. Ltd, and fertilisers for tobacco were provided by Linyi Qingfengnian Agricultural Materials Co. The specific fertiliser situation is shown in **Table 1**.

**Table 1:** Fertilisers used in the experiment.

Type of fertiliser	Formulations	Technical indicators
Fertilisers for tobacco	Granulated	N:P: K=10:10:20
Calcium superphosphate (Ca(OH)2)	Granulated	N:P: K=0:12:0
Potassium sulphate	Granulated	N:P: K=0:0:50
Calcium ammonium nitrate for agriculture	Granulated	N:P: K=15:0:0
Potassium fulvic acid	Granulated	Source fulvic acid≥50.0%, N:P: K=0:0:12

### 2.2 Experimental design

A one-way randomised block design was used for the potting experiment, with six treatments, namely: CK (no fertiliser), NPK (Fertilisers for tobacco 5.85 g/kg), T1 (potassium fulvic acid fertiliser 3.45 g/kg), T2 (potassium fulvic acid fertiliser 4.65 g/kg), T3 (potassium fulvic acid fertiliser 5.85 g/kg), T4 (potassium fulvic acid fertiliser 7.05 g/kg), 10 pots of each treatment, each with one tobacco plant, with a diameter of 34 cm, height of 25 cm, and each with 15 kg of soil. T3 (potassium fulvic acid fertiliser 5.85 g/kg), T4 (potassium fulvic acid fertiliser 7.05 g/kg) 10 pots for each treatment, 1 tobacco plant per pot, the pots were filled with a mixture of soil and fertiliser, with 70% as the basal fertiliser and 30% as the supplementary fertiliser. After transplanting, the plant was managed according to the water and fertiliser management measures for tobacco plant growth, and the fertiliser application rate was converted from field and pot. N:P:K (1:1.5:3) applications, i.e., the same rates as those used for local on-farm crop management, were carried out between the control and treatment groups to ensure that any changes observed between these groups would not be attributed to differences in the application of these nutrients. The exact amount of fertiliser applied is shown in **Table 2**.

**Table 2:** Fertiliser application rate (g/kg) for each treatment in a pot experiment.

Fertiliser	Treatment					
	CK	NPK	T1	T2	T3	T4
Potassium fulvic acid	0	0	3.45	4.65	5.85	7.05
Fertilisers for tobacco	0	5.85	0	0	0	0
Potassium sulphate	0	1.17	2.7	2.4	2.1	1.8
Calcium superphosphate (Ca(OH)2)	0	2.4	7.35	7.35	7.35	7.35
Calcium ammonium nitrate for agriculture	0	0	3.9	3.9	3.9	3.9

### 2.3 Measurement items and methods

#### 2.3.1 Soil sampling

After 65 d of planting the tobacco plants, 0-20 cm of soil was randomly sampled throughout the test site using the five-point composite method, and the samples were passed through a 2-mm sieve to remove plant residues and gravel, and then divided into two parts: the first part was air-dried to analyse the soil enzyme activities and soil physicochemical properties; and the second part was refrigerated at -80°C for soil microbial DNA analyses. The soil samples were kept at Shandong Agricultural University.

#### 2.3.2 Soil physico-chemical properties and enzyme activities

pH measurements were carried out using a pH meter/potentiometer (PHBJ-260 portable pH meter, 0.01 pH resolution; Shanghai Precision Scientific Instruments, Shanghai, China). Potassium dichromate heating method was used to determine the soil organic matter content. Soil quick-acting nitrogen (AN), quick-acting phosphorus (AP) and quick-acting potassium (AK) were determined by alkali hydrolysis diffusion method, molybdenum-antimony antimony colorimetric method and ammonium acetate leaching-flame photometer method, respectively. Soil catalase (S-CAT), soil sucrase (S-SC), and soil urease (S-UE) activities were determined using enzyme activity kits (Beijing Solebo Technology Co., Ltd.), following the manufacturer’s instructions.

#### 2.3.3 Disease incidence analysis

According to the tobacco disease classification and survey method (GB/T23222-2008), the occurrence of tobacco virus disease and tobacco soil-borne diseases (tobacco black shin disease and tobacco root black rot) in each treatment was investigated 65 days after transplanting. The incidence rate and disease index of each treatment were calculated.


$$\begin{array}{c} \text{Incidence}\;\text{rate}\;=\;[\text{number}\;\text{of}\;\text{diseased}\;\text{leaves}\;(\text{plants})/\\ \;\;\;\text{total}\;\text{number}\;\text{of}\;\text{investigations}\;(\text{plants})]\times 100\% \end{array}$$

$$\begin{array}{c} \text{Disease}\;\text{index}\;=\sum (\text{number}\;\text{of}\;\text{disease}\;\text{levels}\\ \;\;\;\times \text{number}\;\text{of}\;\text{plants}\;\text{at}\;\text{that}\;\text{level})\;/\\ \;\;\;\;(\text{number}\;\text{of}\;\text{highest}\;\text{disease}\;\text{levels}\\ \;\;\;\times \text{total}\;\text{number}\;\text{of}\;\text{plants}\;\text{surveyed})\times 100 \end{array}$$

$$\begin{array}{c} \text{Control}\;\text{effect}\;=\;[(\text{control}\;\text{disease}\;\text{index}\\ \;\;\;\;-\;\text{treatment}\;\text{disease}\;\text{index})/\text{control}\;\text{disease}\;\text{index}]\\ \;\;\;\;\times 100\% \end{array}$$

#### 2.3.4 Tobacco plant growth

Twenty tobacco plants were randomly selected for each treatment, and the plant height, leaf length, leaf width, stem circumference, and effective number of leaves were measured with a tape measure and digital electronic vernier calipers, respectively. The net photosynthetic rate, transpiration rate, stomatal conductance, and intercellular carbon dioxide concentration of leaves were measured using a 3051D plant photosynthesis tester (Zhejiang Top Instrument Co., Ltd.). Chlorophyll content and root vigour were determined using the corresponding test kits (Beijing Solebo Technology Co., Ltd.). Using an electronic scale, the dry and fresh weights of the tobacco plants were weighed. Potassium content in 65d tobacco was determined using a flame atomic absorption spectrophotometer.

### 2.4 Data analysis methods

#### 2.4.1 Soil DNA extraction, PCR amplification and high-throughput gene sequencing

The collected soil samples were handed over to Beijing Baimike Biotechnology Co. Ltd. to extract soil DNA, the extracted DNA was amplified by PCR, and the amplified product was purified, quantified and homogenised to form sequencing library (SMRT Bell), the constructed library was firstly subjected to library quality control, and the library which had passed the quality control was sequenced by PacBio-SSequel, and the offline data of PacBio was exported, and the Raw CCS sequence data were obtained by barcode identification of CCS sequences using lima v1.7.0 software. CCS sequences were identified by barcode using lima v1.7.0 software to obtain Raw CCS sequence data; primer sequences were identified and removed by cutadapt 1.9.1 software and length filtered to obtain Clean CCS sequences with no primer sequences; and then chimeric sequences were identified and removed by UCHIME v4.2 software to obtain valid CCS sequences. Valid CCS sequences were obtained by removing chimeric sequences by UCHIME v4.2 software. Valid CCS sequences were clustered using Usearch software with 97.0% similarity to obtain OTUs; using the SILVA Ribosomal RNA Database (SILVA) as a reference database, sequences were classified and annotated using a simple Bayesian classifier combined with a comparative approach to obtain taxonomic information for each feature, and then the species were obtained at each level (phylum, class, order, Family, etc.) taxonomic information. Taxonomic information can be obtained for each feature and then the community composition of each sample can be counted at each level (phylum, order, order, family, genus, species) and further analyses of α-diversity, β-diversity and significant species differences and correlations can be carried out to explore the differences between soil samples.

#### 2.4.2 Data-processing analyses

All the experimental data, which were collected in three replications, were processed through Microsoft Office Excel 2019 and SPSS 25.0 software for statistical analysis of data on agronomic traits, biomass, photosynthetic rate, chlorophyll, root indexes, soil physicochemical, soil enzyme activity and potassium content of tobacco plants, using Duncan’s test for the Duncan’s test of significance and correlation analysis.

For soil microbial community structure, Alpha diversity was analysed for between-group variability based on Student’s test for rhizosphere soil samples at the OUT level, and Beta diversity for rhizosphere soil samples was analysed for between-group ANOVA using the non-metric multidimensional scaling (NMDS) method based on the Bray-curtis distance algorithm. The relationships between soil physicochemical, soil enzyme activities and soil microorganisms were analysed using redundancy analysis (RDA).

## 3 Results

### 3.1 Effect of different fertiliser treatments on soil mention chemical parameters

**Table 3** shows the physicochemical properties of soil on the 65th day after transplanting, each fertiliser treatment made the soil physicochemical indexes have different degrees of improvement, T2 treatment on the soil of organic matter, quick-acting phosphorus, quick-acting potassium, alkaline dissolved nitrogen increase reached the highest, the increase of 22.04%, 96.21%, 381.79%, 43.12%, respectively, the T2 treatment compared to the CK treatment pH increased by 25.43%. The results showed that the soil nutrients of tobacco plants showed a tendency of increasing firstly and then decreasing with the increase of the application of potassium fulvic acid, and the application of appropriate amount of potassium fulvic acid could promote the increase of soil nutrients of tobacco plants.

**Table 3:** Effects of different fertilisation treatments on soil physicochemical properties.

Treatment	OM (g/Kg)	AP (mg/Kg)	AK (mg/Kg)	N (mg/Kg)	pH
CK	15.47 ± 0.26d	62.01 ± 0.92f	71.65 ± 2.19f	38.96 ± 0.81c	5.91 ± 0.07e
NPK	17.28 ± 0.57b	90.51 ± 0.18e	83.28 ± 1.26e	42.23 ± 0.2bc	6.26 ± 0.03c
T1	17.81 ± 0.01b	116.95 ± 0.81b	143.3 ± 4.37d	45.5 ± 3.05b	6.38 ± 0.03b
T2	18.88 ± 0.13a	121.67 ± 1.08a	291.2 ± 6.04a	55.76 ± 2.91a	6.65 ± 0.06a
T3	17.66 ± 0.26b	107.86 ± 0.47c	265.6 ± 8.47b	45.38 ± 0.53b	6.45 ± 0.04b
T4	16.53 ± 0.52c	104.67 ± 0.18d	163.58 ± 1.65c	41.06 ± 0.4c	6.00 ± 0.04d

Lowercase letters Represents an annotation of the significance level of the data.

### 3.2 Effect of different fertilisation treatments on soil enzyme activities

**Figure 1:**
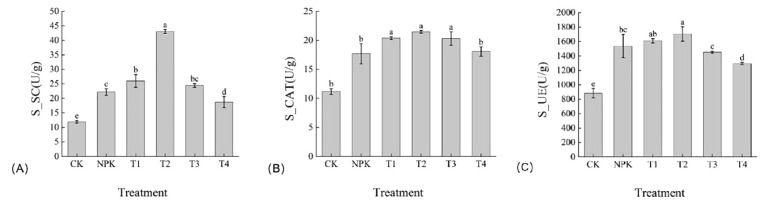
Enhancement of soil enzyme activities by Potassium fulvic acid. **(A)** Detection of soil sucrase(S-SC) enzyme activities in control plants and Potassium fulvic acid-treated plants **(B)** Detection of soil peroxidase(S-CAT) enzyme activities in control plants and Potassium fulvic acid-treated plants **(C)** Detection of soil urease(S-UE) enzyme activities in control plants and Potassium fulvic acid-treated plants (u/g is the international unit of enzyme viability. It refers to the amount of enzyme required to convert 1 micromole of substrate, or 1 micromole of the relevant group in the substrate, in 1 minute under specific conditions). Lowercase letters Represents an annotation of the significance level of the data.

The enzymatic activity results are summarized in **Figure 1**. Soil sucrase(S-SC) is an enzyme secreted by microorganisms, mainly due to the decomposition of sucrose in the soil, which can help plants to absorb sucrose, thus promoting the growth and development of plants. S-SC activity increased significantly in the treatment groups compared with those in the control group, and the T2 treatment had the highest sucrase content, which was 66.51%, 76.07%, 94.01%, 129.85%, and 262.07% higher than that of T1, T3, NPK, T4, and CK, respectively (**Figure 1A**). Soil peroxidase(S-CAT) can promote chemical reactions in soil and improve soil fertility and ecology. The S-CAT activity of T2 was higher than the other treatments in each treatment group, but there was no significant difference with T1,T3 (**Figure 1B**). Soil urease(S-UE) is involved in the decomposition process of urea in the soil, by converting urea to nitrogen, making urea available for plant use. Similarly, the S-UE activity of T2 treatment was higher than the other treatments and was 5.20%, 5.61%, 18.71%, 21.32%, 91.69% higher than T1, NPK, T3, T4 and CK treatments, respectively. The results showed that the soil enzyme activities of tobacco plants showed a tendency of increasing and then decreasing with the increase of the application amount of potassium fulvic acid, and the application of appropriate amount of potassium fulvic acid could promote the increase of soil enzyme activities of tobacco plants.

### 3.3 Effect of different fertiliser treatments on soil rhizosphere microbial diversity

#### 3.3.1 High-throughput sequence quality control data

The collected rhizosphere soil samples were sequenced, and the sequencing results showed that for the bacterial community structure, a total of 1456251 pairs of Reads were obtained from the 18 soil samples, and a total of 1329250 Clean Reads were generated after double-ended Reads QC, splicing, and at least 72528 Clean Reads were generated for each sample, with an average of 73847 For fungal community structure, a total of 1489256 pairs of Reads were obtained from 18 soil samples, and a total of 1327954 Clean Reads were generated from double-ended Reads QC, splicing, and generating at least 61,621 Clean Reads per sample and 73,775 Clean Reads on average. Based on these sequences clustered at 97.0% similarity level, a total of 18765 bacterial OTUs and 5437 fungal OTUs were obtained.

#### 3.3.2 Distribution of relative abundance of rhizosphere microorganisms in different fertiliser treatments

##### 3.3.2.1 Based on the relative abundance of rhizosphere microorganisms at the gate level

**Figure 2:**
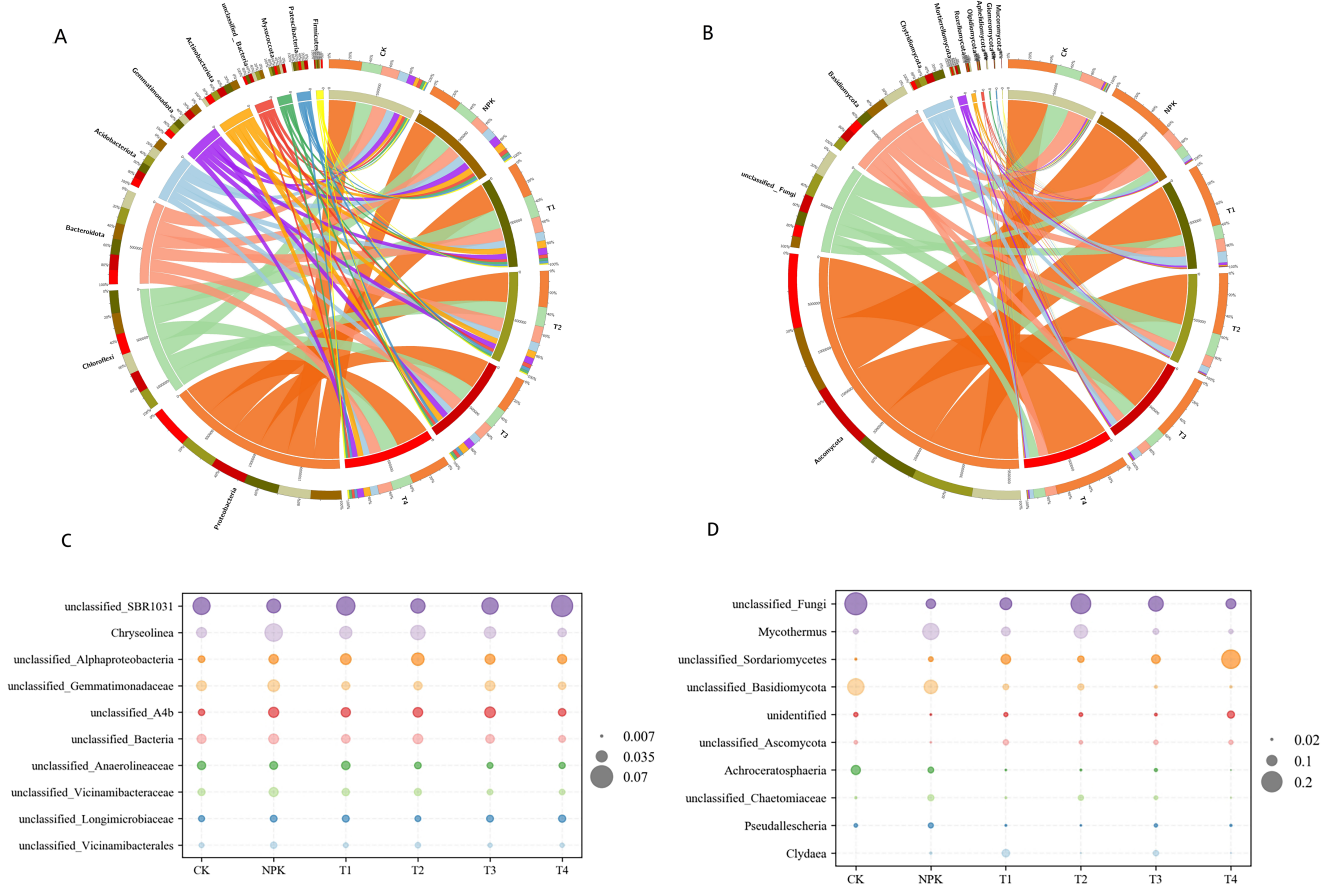
Distribution of relative abundance of rhizosphere microorganisms. Species abundance at the bacterial **(A)** and fungal **(B)** phylum level (Types of colours represent different phylums, The width of the stripes represents the percentage of processing they occupy). Horizontal species abundance of bacterial **(C)** and fungal **(D)** genera under different fertilisation treatments.

As shown in **Figure 2A**, after comparison with the database, the distribution of the Top 10 species of soil sample bacteria at the phylum level can be found, Proteobacteria, Chloroflexi, Bacteroidota, Acidobacteriota, Gemmatimonadota, Actinobacteriota, etc. are the dominant phyla. Proteobacteria: T4 > T2 > T3 > T1 > CK > NPK, with T1, T2, T3 and T4 treatments increasing by 0.03%, 0.1%, 0.07% and 0.2%, respectively, compared with the CK treatment; Chloroflexi: T1 > CK > NPK > T4 > T2 > T3, with the exception of the T1 treatment, which had an increase, and the T2 and T3 treatments decreased in abundance by 0.06% and 0.06%, respectively, compared with CK; Bacteroidota: CK>T2>NPK>T1>T3>T4, compared with the abundance of CK treatments, the abundance of all treatments decreased; Acidobacteriota: NPK>CK>T1>T2>T3>T4, compared with the abundance of CK treatments, the NPK treatments increased by 0.11%, while T1, T2, T3 and T4 treatments decreased by 0.125%, 0.125%, 0.29% and 0.29%, respectively; Gemmatimonadota: T2 > NPK > T3 > T1 > T4 > CK, with the abundance of all the treatments increased, among which, the abundance of T2 treatment was the highest; Actinobacteriota. NPK > T1 > T3 > T2 > T4 > CK, and the relative abundance of each treatment increased compared with the CK treatment. It can be seen that each fertilisation treatment affected the relative abundance of bacterial community, among which, T2 treatment increased the relative abundance of Gemmatimonadota. Gemmatimonadota are reported to play an important role in the material cycle and energy flow. They are able to utilise a wide range of organic matter as a source of carbon and energy and participate in the degradation process of organic matter. This helps to maintain the balance and stability of the ecosystem (Mujakić et al., 2023).

As shown in **Figure 2B**, after comparison with the database, the distribution of Top10 species of soil sample fungi at the phylum level can be found, with Ascomycota, unclassified_Fungi, Basidiomycota, and Chytridiomycota as the dominant phyla. Ascomycota: T4 > NPK > T3 > T1 > T2 > CK, and the abundance of each fertiliser treatment increased compared to the CK treatment, by 0.54%, 0.40%, 0.33%, 0.30% and 0.26%, respectively; Basidiomycota: CK > NPK > T1 > T3 > T4 > T2, and the relative abundance of each fertilisation treatment was reduced by 0.1%, 0.83%, 1%, 1% and 1.2%, respectively, compared with CK treatment, of which the T2 treatment had the lowest relative abundance; Chytridiomycota: T1 > T2 > T3 > T4 > NPK > CK, and the abundance of each fertiliser treatment increased compared to the CK treatment, by 10%, 6%, 6%, 3% and 3%, respectively; Mortierellomycota: T2 > T3 > T1 > T4 > CK > NPK, and fulvic acid decreased by 0.1%, 0.83%, 1%, 1% and 1.2%, respectively, compared to the CK treatment, higher relative abundance in potassium fulvic acid treatment, among which, T2 treatment was the highest. It can be seen that each fertilisation treatment affected the relative abundance of fungal communities, in which each fertilisation treatment increased the relative abundance of the pathogenic fungus Chytridiomycota, but also increased the relative abundance of the beneficial fungus Mortierellomycota, with the highest relative abundance in the T2 treatment.

##### 3.3.2.2 Based on the relative abundance of rhizosphere microorganisms at the genus level

As shown in **Figure 2C**, at the bacterial genus level, the dominant genera in the soil samples of each treatment were mainly *unclassified_SBR1031*, *Chrysolinea*, *unclassified_Alphaproteobacteria*, *unclassified_Gemmatimonadaceae*, *unclassified _A4b*, *unclassified_Bacteria* and so on. Among them, *unclassified_Alphaproteobacteria, unclassified_Gemmatimonadaceae and unclassified_Gemmatimonadaceae* abundance increased in all fertilised treatments compared to the CK treatment, the highest comparative abundance was found in the T2 treatment.

As shown in **Figure 2D**, at the level of fungal genera, the dominant genera in the soil samples of each treatment were mainly *unclassified_Fungi*, *Mycothermus*, *unclassified_Sordariomycetes*, *unclassified_Basidiomycota*, and *Fusarium*. Among them, *Fusarium*: T3>T4>T1>NPK>CK>T2, and the relative abundance of Fusarium spp. was the lowest in T2 treatment.

#### 3.3.3 Analysis of microbial Alpha diversity in rhizosphere soils with different fertilisation treatments

**Figure 3:**
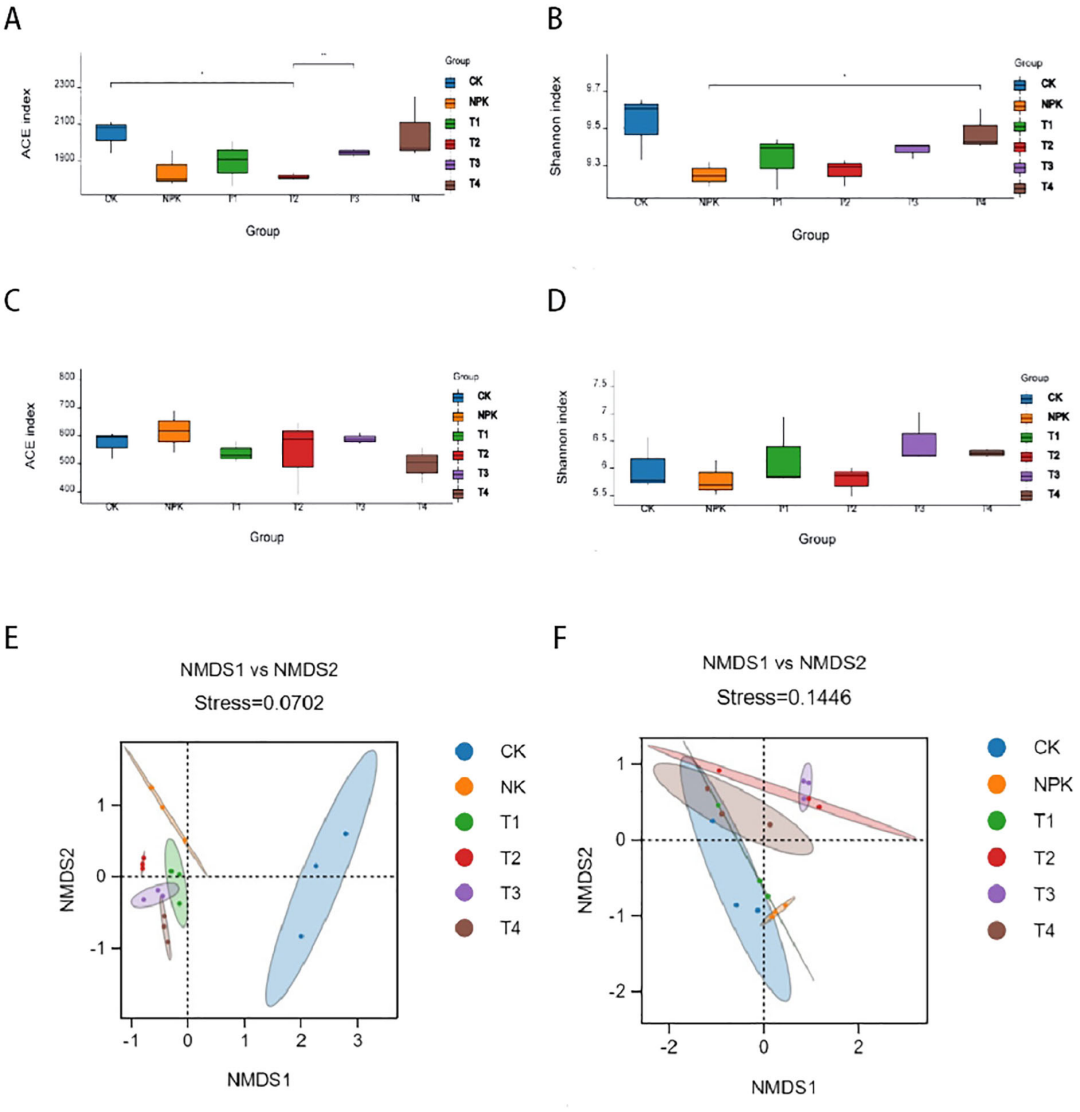
Analysis of microbial diversity in inter-root rhizosphere soils. Analysis of Alpha diversity of bacteria **(A, B)** and fungi **(C, D)** under different fertilisation treatments. Beta diversity analysis of bacteria **(E)** and fungi **(F)** under different fertilisation treatments.

Alpha diversity reflects the species abundance and species diversity of the samples, where Shannon and Simpson indices are used to measure the species diversity the larger the value, the higher the species diversity of the samples; Chao1 and Ace indices are used to measure the species abundance, i.e., the number of species. As can be seen in **Figures 3A-D**, in the bacterial community, species abundance and diversity decreased for each fertilisation treatment. In the fungal community, the Shannon index increased for each potassium fulvic acid treatment, indicating that the diversity of the fungal community increased in potassium fulvic acid treatments, all of which increased the diversity of the fungal community. The comprehensive analysis indicated that the application of potassium fulvic acid significantly changed the composition of the rhizosphere soil microbial community of tobacco plants.

#### 3.3.4 Beta diversity analysis of rhizosphere soil microorganisms in different fertilisation treatments

In order to compare the degree of similarity that exists in terms of species diversity in different samples, the use of NMDS analysis (non-metric multidimensional scaling) can reveal differences in microbes between or within groups, and in the present study NMDS analysis based on Bray-Curtis variability reflected the β-diversity of the soil microbes without use of treatments. As shown in **Figure 3E**, in the rhizosphere soil bacterial microbial community, there was a significant separation (stress: 0.0702) between the control and fertiliser treatments, two kinds of significant separation in the NMDS1 direction, and the T2 treatment was farthest away from the control, with no significant difference in the NMDS2 direction. The fertilised treatments were clustered in the graph with intersecting distances between the two points, indicating that the microbial communities differed little between the fertilised treatments. The separation between the groups indicated that the fertilisation treatments had a significant effect on the bacterial community at the same time, among which, the effect of T2 treatment was more significant. Comprehensive analysis of potassium fulvic acid can change the composition of soil bacterial community, and there are obvious differences between different dosages of potassium fulvic acid.

The NMDS analysis of the fungal community, shown in **Figure 3F**, indicated that there was no clustering between the control and the other treatments (stress: 0.1446). The T3 treatment was furthest away from the control in the NMDS1 direction. There was no significant separation between the fertiliser treatments and the control, indicating that the fertiliser treatments had no significant effect on the fungal microbial community.

#### 3.3.5 Analysis of differences between rhizosphere soil microbiomes of different fertilisation treatments

**Figure 4:**
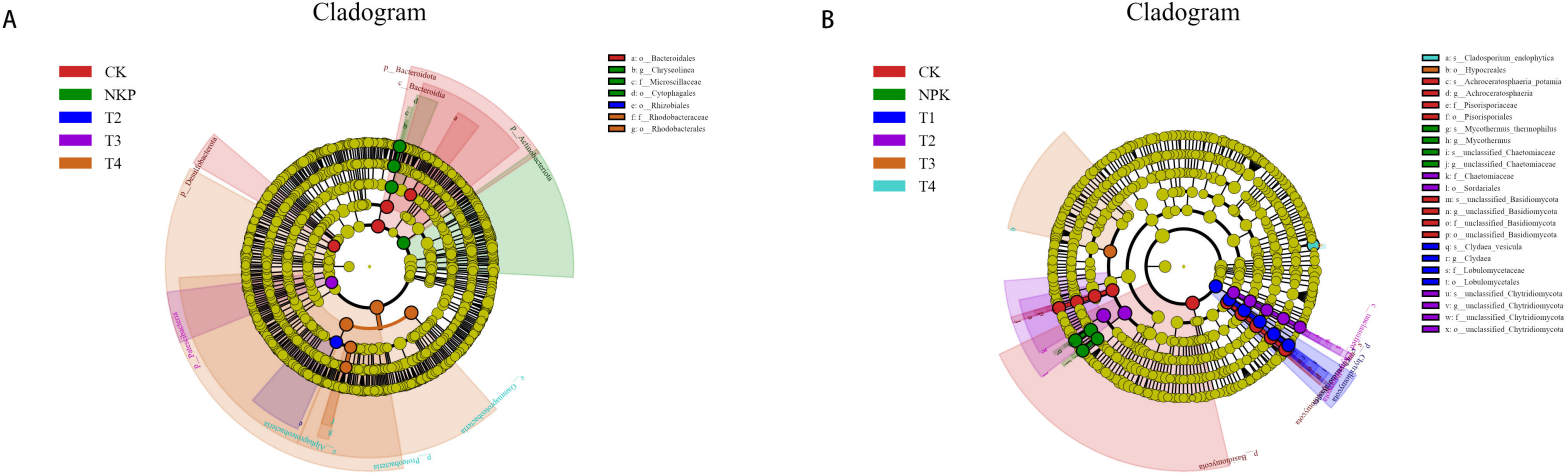
Analysis of bacterial LEfSe **(A)** and fungal LEfSe **(B)** in different fertiliser treatments.

LEfSe software was used to detect significant differences in bacterial and fungal abundance at the phylum and genus levels. As can be seen in **Figure 4**, the results showed that at the bacterial phylum level, 2, 1, 1 and 1 biomarkers were found in CK (Bacteroidota and Desulfobacterota), conventional fertilisation (Acidobacteriota), T3 (Patescibacteria) and T4 (Proteobacteria) treatments, respectively. At the fungal phylum level, 1 biomarker was found for each of the CK (Basidiomycota) and T1 (Chytridiyomycota) treatments. LEfSe analyses showed that at the bacterial phylum level, only 1 biomarker was found for conventional fertilisation. At the fungal genus level, 2, 2, 1, 1, 1, 1 and 1 biomarkers were found for CK, conventional fertilisation, T1, T2 and T4, respectively. This is in agreement with the results of relative abundance of species, which suggests that each fertilisation treatment had an effect on the soil microbial community structurally.

### 3.4 Analysis of correlations between rhizosphere soil microorganisms

Network diagram-based analyses can obtain information on the coexistence of species in environmental samples and obtain information on the interactions and important patterns of species in the same environment to further explain the mechanisms shaping phenotypic differences between samples. To explore ecological interaction patterns between fungi and bacteria in rhizosphere communities, a microbial community correlation network diagram for bacteria and fungi was developed based on strong correlations at the genus level, showing the top 50 genera with the highest correlations.

**Figure 5:**
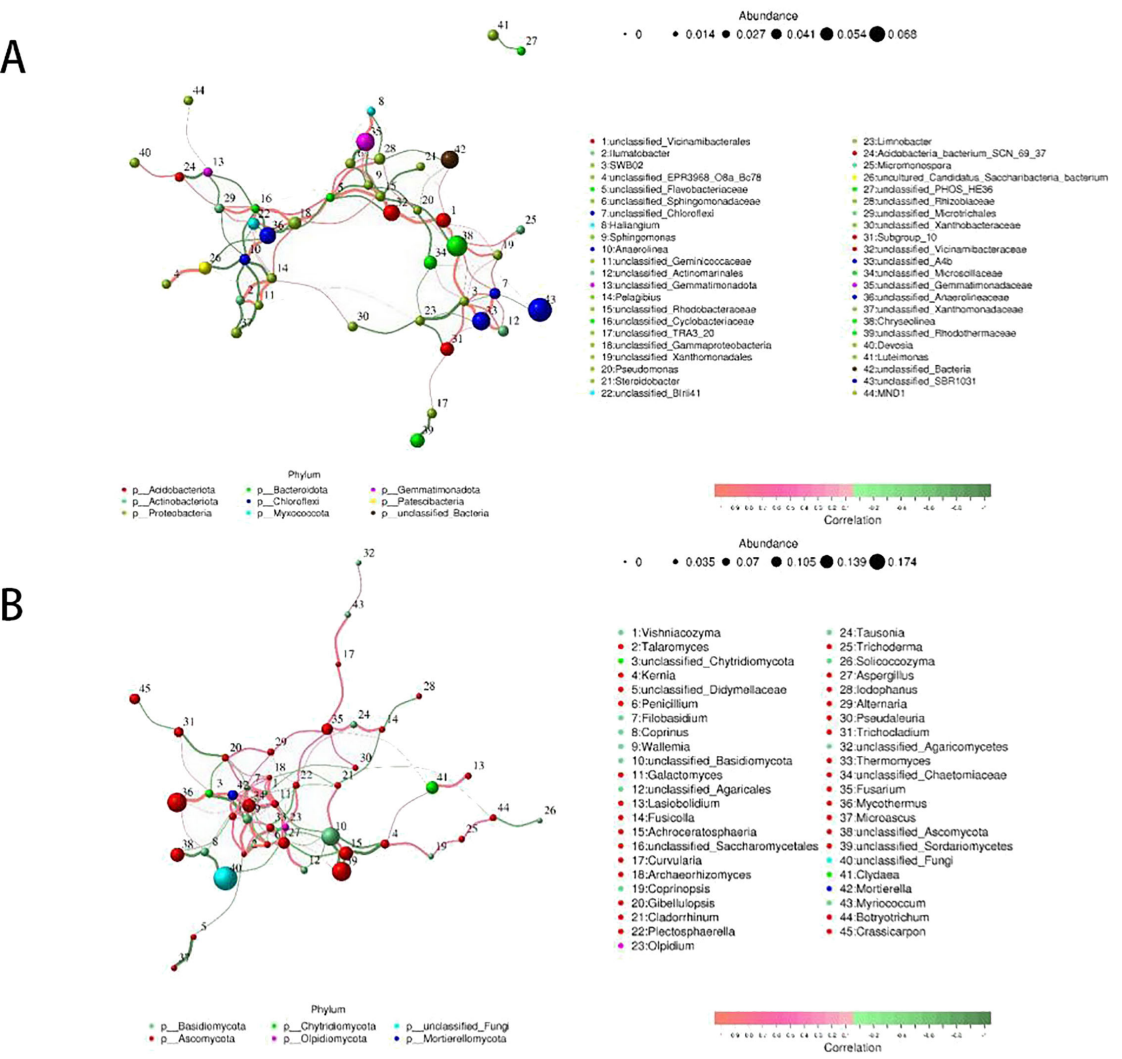
Correlation network of soil rhizosphere bacteria **(A)** and fungi **(B)**.

From **Figures 5A, B**, it can be seen that the positive correlation between bacterial and fungal communities is higher than the negative correlation, and the network complexity of bacterial communities is higher than that of fungal communities. From **Figure 5A**, it can be seen that in the bacterial community network, most of the nodes belong to Proteobacteria, Chloroflexi, Acidobacteriota and Bacteroidota, the strain numbered 43, the relative abundance is larger, the figure with the strain numbered 43 belongs to Chloroflexi, indicating that fertiliser treatments make the Chloroflexi’s abundance increased, and Chloroflexi in turn interacted with other phyla and had an effect on the structure of soil rhizosphere microbial communities.

As can be seen in **Figure 5B**, most of the nodes in the fungal community network belonged to Ascomycota and Basidiomycota, which is consistent with the performance of the relative abundance graph of the fungal community, with the strain numbered 40, which has a greater relative abundance and a stronger correlation with Basidiomycota. This network diagram suggests that microbial interactions and fertiliser incorporation changed the structure of the soil microbial community.

### 3.5 Analysis of correlations between rhizosphere microbial communities and environmental factors

#### 3.5.1 RDA analysis of rhizosphere microbial communities and soil physico-chemical traits

**Figure 6:**
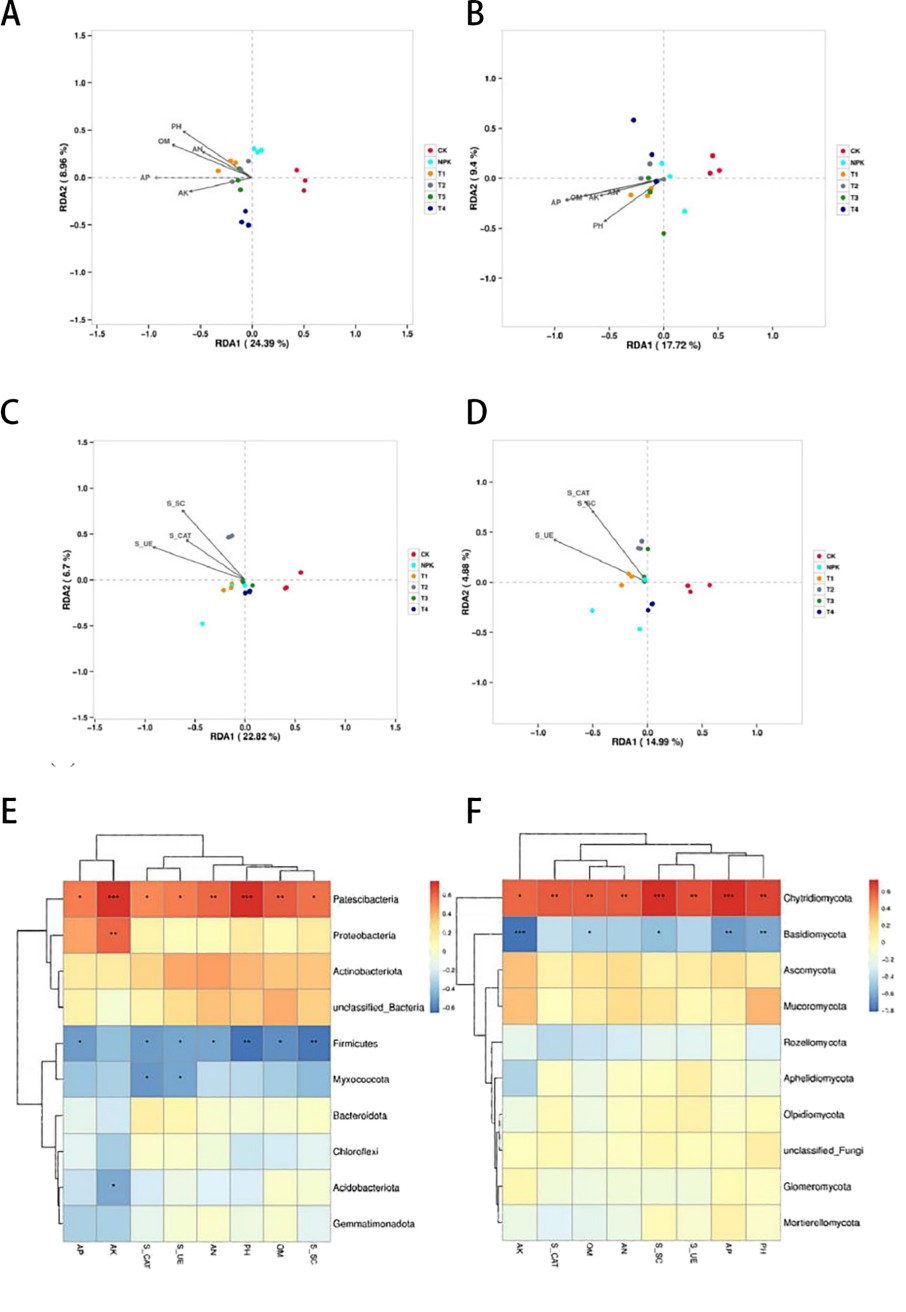
RDA analysis of soil physicochemical properties at bacteriophyla level **(A)** and mycophyla level **(B)** under different fertilisation treatments. RDA analysis of soil enzyme activity at bacteriophyla level **(C)** and mycophyla level **(D)** under different fertilisation treatments. Heat map analysis of the correlation of soil microorganisms bacterium **(E)** and fungi **(F)** with soil properties at gate level (* indicates the strength of the correlation). ** And *** denote significant correlation strength at p <0.05.

To identify potential environmental drivers, the relative abundance of bacterial and fungal communities was correlated with environmental factors using RDA analysis, and the spatial relationship between the sample points and the environmental factor vectors in the RDA analysis plots, reflecting the correlation between them. To identify potential environmental drivers, the relative abundance of bacterial and fungal communities was correlated with environmental factors using RDA analysis. As can be seen in **Figure 6A**, RDA analysis indicated that among the bacterial communities, the abundance of AN, AP, AK, pH and SOM in the soil had a significant effect on the microbial community composition, with AN being the most important factor. samples from the T1 and T2 treatments showed a significant positive correlation with AN, AP, AK, pH, and SOM, which had stronger biological correlations. samples from the CK treatment were significantly and physically correlated with soil physical and chemical negative correlation. As shown in **Figure 6B**, RDA analysis indicated that among the fungal communities, the abundance of AN, AP, AK, pH and SOM in soil had a significant effect on the composition of microbial communities, with AN being the most important factor. samples from the T1, T2 and T3 treatments were positively correlated with AN, AP, AK, pH and SOM with strong correlation, while those from the CK and NPK treatments were significantly negatively correlated with soil physico-chemicals. were significantly negatively correlated.

#### 3.5.2 RDA analysis of rhizosphere microbial communities and soil enzyme activities

RDA analyses of soil enzyme activities and environmental factors explained the total changes in the structure of soil bacterial and fungal communities, respectively. As can be seen in **Figure 6C**, the RDA analyses showed that, in the bacterial community, the activities of soil catalase, urease, and sucrase had a significant effect on the composition of the microbial community, where soil catalase had a stronger effect on the community. The samples from the CK treatments showed a significant negative correlation with enzyme activity. From **Figure 6D**, RDA analysis showed that CK treated samples were significantly negatively correlated with enzyme activities in fungal community.

#### 3.5.3 Heat map analysis of correlation between rhizosphere microbial communities and environmental factors

The correlation heatmap (Heatmap) is a presentation of the correlation relationship between species and environmental factors, and the correlation heatmap shows the top 10 phyla of bacterial and fungal communities. As can be seen in **Figure 6E**, the correlation heatmap shows that the dominant bacterial phyla Proteobacteria, Actinobacteriota, Myxococcota, Firmicutes, and Patescibacteria were significantly affected by soil factors, Proteobacteria, Patescibacteria and Actinobacteriota were positively correlated with soil environmental factors, and Myxococcota, Firmicutes, Microbacterium wartyi and Desulfovibrio thermophilus were significantly negatively correlated with soil environmental factors. As can be seen from **Figure 6F**, the dominant fungal phyla Ascomycota, Basidiomycota, Rozellomycota and Chytridiomycota were significantly affected by soil factors, Basidiomycota, Rozellomycota, Wormy mildew and Mycobacterium phyla were significantly negatively correlated with soil environmental factors and Chytridiomycota was significantly positively correlated with soil environmental factors.

As can be seen in **Figure 6E**, in the bacterial community, the abundance of Patescibacteria, Proteobacteria, and Acidobacteriota was significantly and positively correlated with the content of soil AN, AP, AK, pH, SOM, S_SC, S_CAT, and S_UE, and more strongly correlated with the distribution of samples in T1 and T2 treatments The abundance of Firmicutes and Myxococcota was significantly and negatively correlated with the content of soil AN, AP, AK, pH, SOM, S_SC, S_CAT and S_UE and more strongly correlated with the sample distribution of the CK treatment. As can be seen in **Figure 6F**, among the fungal communities, the abundance of Chytridiomycota, Ascomycota, and Mucoromycota was significantly and positively correlated with soil AN, AP, AK, pH, SOM, S_SC, S_CAT, and S_UE contents, while Basidiomycota abundance was significantly and positively correlated with soil AN, AP, AK, pH, SOM, S_SC, S_CAT and S_UE contents were significantly negatively correlated.

It is hypothesised that Patescibacteria, Proteobacteria, and Actinobacteriota in the bacterial community may have some promoting effect on plant growth. Chytridiomycota and Basidiomycota, which are potential pathogenic bacteria in the fungal community, may have a certain inhibitory effect on plant growth.

### 3.6 Effect of different fertiliser treatments on the morphology of tobacco plants

#### 3.6.1 Effect of different fertiliser treatments on agronomic traits of tobacco plants

In order to assess the effect of different concentrations of potassium fulvic acid on the growth and development of tobacco plants, plant height, stem circumference, number of leaves, maximum leaf length, and maximum leaf width during the growth and development period of tobacco were determined. For agronomic traits during the growth and development period, the results of the experiment showed that there were no significant differences between the treatments at the 15th and 30th days after transplanting, and all the fertiliser treatments significantly increased the height, stem girth, number of leaves, maximum leaf length, and maximum leaf width of the tobacco plants compared to the control group. Agronomic traits differed significantly among treatments from the 45th d after transplanting. The plant height of T2 and T3 treatments showed an increasing trend compared to conventional fertiliser application, the maximum leaf length of T2 and T3 treatments was the best compared to conventional fertiliser application, the maximum leaf width of T2 treatment was the best compared to conventional fertiliser application at 60d, the stem girth of T2 treatment showed an increasing trend compared to conventional fertiliser application, and the effective number of leaves of T2 and T3 treatments was continuously increasing compared to conventional fertiliser application (**Table 4**). The results showed that too much or too little potassium fulvic acid could not promote the growth and development of tobacco plants well, and the appropriate amount of potassium fulvic acid could effectively improve the growth and development of tobacco plants.

**Table 4:** Comparison of agronomic traits of different fertiliser treatments for the same number of growing days.

Time	Treatment	Plant height cm	Maximum leaf length cm	Maximum blade width cm	Stem girth cm	Effective blade number
Day 15 after transplanting	CK	5.37 ± 0.65c	17.45 ± 3.80a	5.78 ± 0.86b	1.93 ± 0.37c	4.00 ± 0.63a
	NPK	5.48 ± 0.74bc	17.03 ± 1.76a	6.67 ± 0.71ab	2.17 ± 0.20bc	4.50 ± 0.55a
	T1	6.07 ± 0.56abc	16.35 ± 3.42a	6.57 ± 1.17ab	2.23 ± 0.39bc	4.67 ± 0.52a
	T2	6.33 ± 0.93ab	18.50 ± 1.39a	7.23 ± 0.70a	2.78 ± 0.12a	4.67 ± 0.52a
	T3	6.82 ± 0.40a	19.28 ± 1.86a	7.35 ± 0.87a	2.42 ± 0.39ab	4.50 ± 0.55a
	T4	6.03 ± 0.88abc	16.13 ± 2.87a	6.62 ± 1.46ab	1.98 ± 0.35c	4.50 ± 0.55a
Day 30 after transplanting	CK	8.83 ± 1.61a	22.77 ± 9.68a	8.73 ± 1.07a	2.23 ± 0.32c	6.00 ± 2.00a
	NPK	10.16 ± 1.76a	27.37 ± 0.81a	11.23 ± 0.25a	2.90 ± 0.36b	7.33 ± 0.58a
	T1	10.67 ± 1.61a	28.30 ± 3.87a	11.03 ± 1.35a	3.17 ± 0.49b	7.33 ± 0.58a
	T2	11.00 ± 1.50a	31.03 ± 2.59a	11.27 ± 1.10a	4.07 ± 0.12a	7.67 ± 0.58a
	T3	10.93 ± 1.48a	29.53 ± 3.23a	11.00 ± 1.50a	3.80 ± 0.20a	7.33 ± 0.58a
	T4	9.10 ± 0.36a	23.23 ± 3.22a	9.17 ± 3.69a	2.77 ± 0.25bc	6.67 ± 0.58a
Day 45 after transplanting	CK	10.00 ± 2.00c	29.23 ± 1.96b	10.93 ± 0.58b	3.73 ± 0.51a	8.33 ± 1.15c
	NPK	16.00 ± 0.50b	38.57 ± 2.21a	13.93 ± 0.40a	4.13 ± 0.47a	10.33 ± 0.58ab
	T1	15.83 ± 2.36b	33.03 ± 2.66b	11.83 ± 2.05ab	4.10 ± 0.72a	9.33 ± 0.58bc
	T2	21.33 ± 1.89a	40.13 ± 2.60a	14.03 ± 1.10a	4.37 ± 0.58a	11.33 ± 0.58a
	T3	19.33 ± 0.29a	38.67 ± 2.40a	13.97 ± 1.22a	4.43 ± 0.21a	11.00 ± 1.00a
	T4	14.50 ± 2.50b	32.60 ± 0.87b	11.50 ± 0.86b	3.93 ± 0.81a	9.33 ± 0.58bc
Day 60 after transplanting	CK	14.43 ± 4.33d	30.53 ± 4.82b	12.73 ± 1.80a	4.20 ± 0.36b	9.33 ± 1.53b
	NPK	26.67 ± 0.58bc	37.33 ± 3.21ab	14.23 ± 0.68a	4.53 ± 0.41b	13.37 ± 0.64a
	T1	28.13 ± 0.98abc	37.30 ± 3.82ab	14.43 ± 2.10a	4.73 ± 0.61ab	14.00 ± 1.73a
	T2	32.90 ± 3.44a	43.40 ± 5.41a	16.73 ± 2.76a	5.47 ± 0.06a	15.00 ± 1.00a
	T3	31.30 ± 2.19ab	40.93 ± 5.08a	14.43 ± 2.68a	4.60 ± 0.20ab	14.33 ± 0.58a
	T4	24.37 ± 2.58c	36.20 ± 3.30ab	14.07 ± 1.91a	4.33 ± 0.81b	13.33 ± 0.58a

Lowercase letters Represents an annotation of the significance level of the data.

#### 3.6.2 Effect of different fertiliser treatments on biomass accumulation in tobacco plants

**Figure 7:**
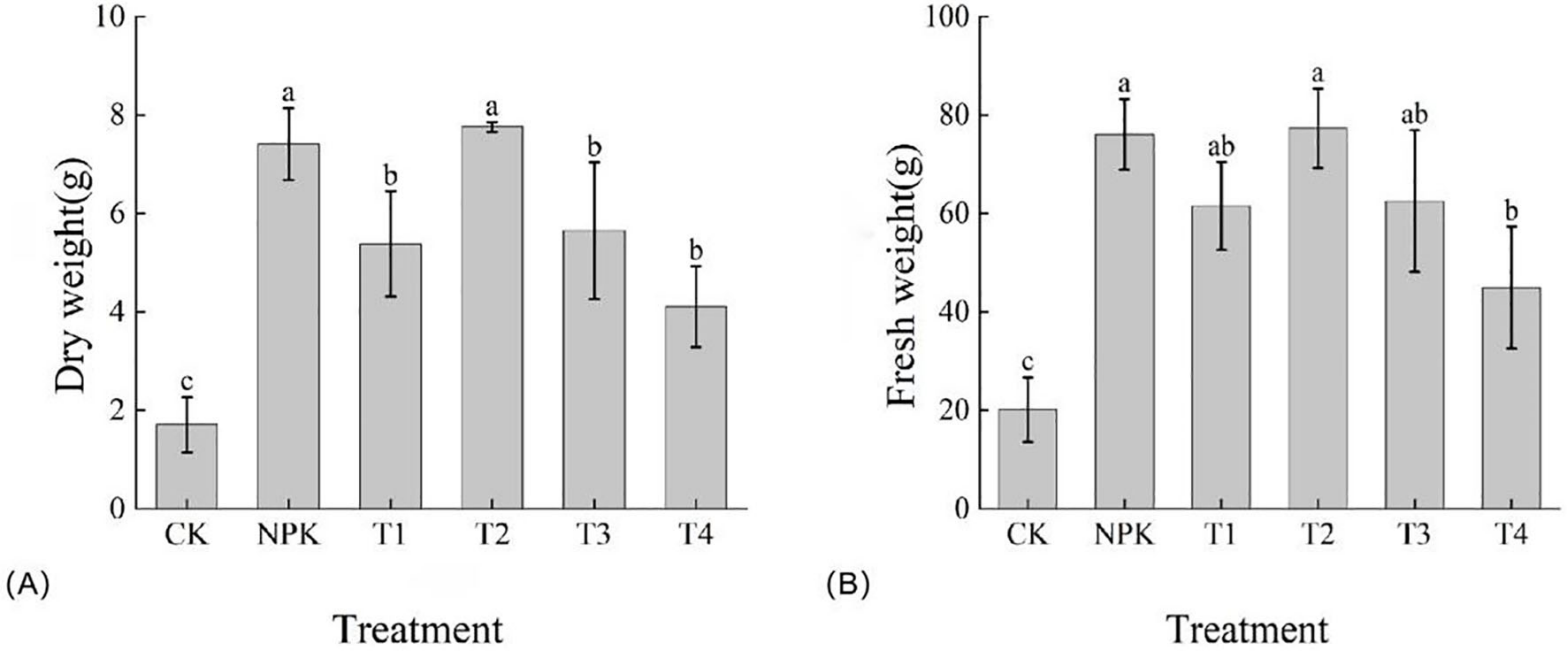
Effects of different fertilisation treatments on the dry weight **(A)** and fresh weight **(B)** of tobacco matter accumulation. Lowercase letters Represents an annotation of the significance level of the data.

As shown in **Figure 7**, the dry weight size of each treatment was T2>NPK>T3>T1>T4>CK, and T2 treatment was higher than the other treatments, with the dry weight reaching 7.76 g. However, there was no significant difference between the T2 treatment and the NPK treatment, and the difference between the T1, T3 and T4 treatments was not significant. The fresh weight of each treatment was T2>NPK>T3>T1>T4>CK, and T2 treatment was higher than other treatments, with fresh weight reaching 77.32 g, but there was no significant difference between T2 and NPK treatments, and no significant difference between T1 and T3 treatments. The fresh weight of the treatments echoed with the dry weight, and collectively, the T2 treatment was superior in terms of tobacco biomass accumulation. The biomass accumulation could be better promoted by applying appropriate amount of potassium fulvic acid.

#### 3.6.3 Effect of different fertiliser treatments on the root system of tobacco plants

##### 3.6.3.1 Effect of different fertilisation treatments on root morphology of tobacco plants

**Figure 8:**
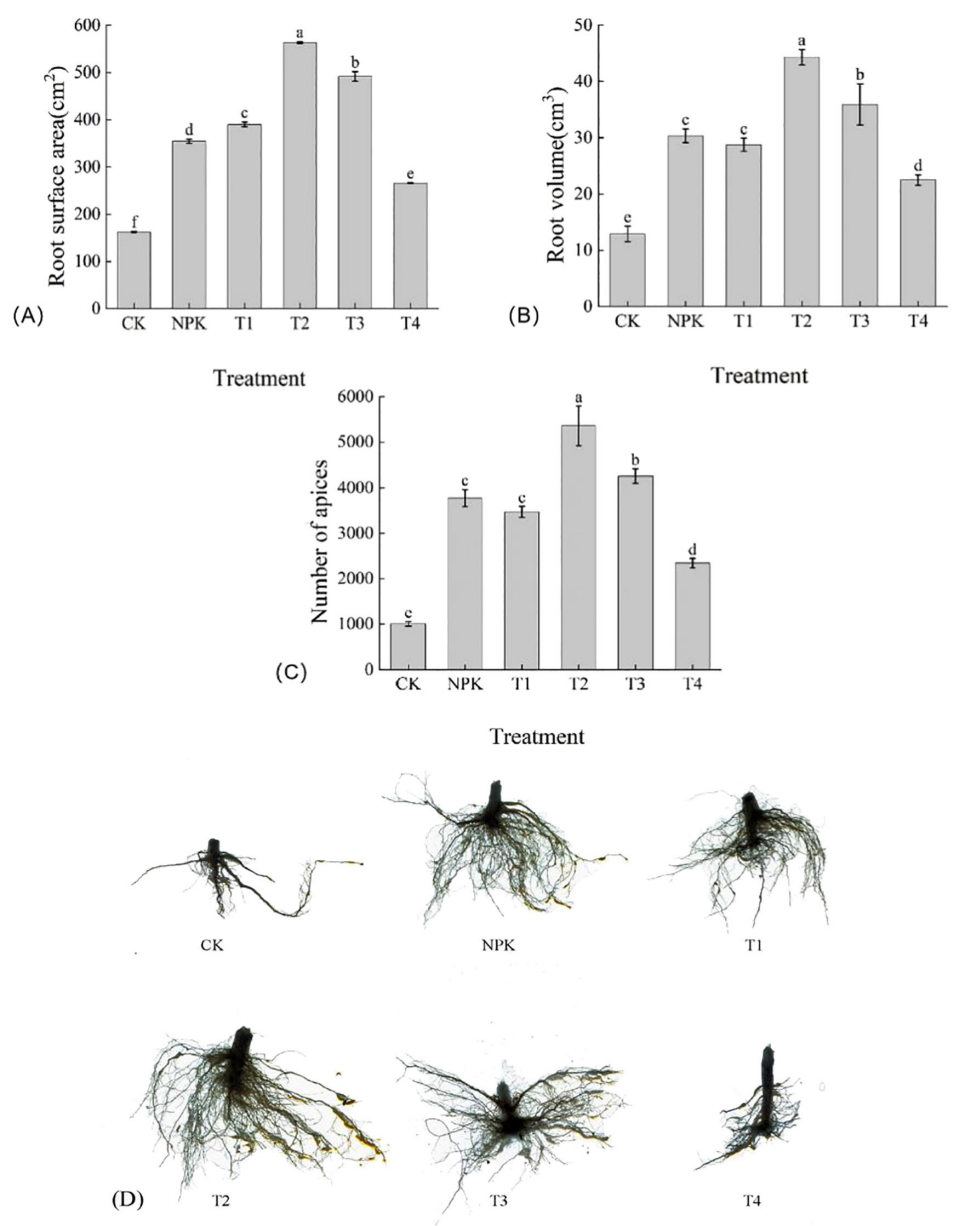
Effects of different fertilisation treatments on root indices (root surface area **(A)**, root volume **(B)** and root tip number **(C)** and root morphology **(D)** of tobacco. Lowercase letters Represents an annotation of the significance level of the data.

As shown in **Figure 8**, root surface area, root volume, root tip number, and root morphology under each fertilisation treatment were greater than those of T4 and CK treatments. The differences in root surface area were significant, with the size of T2>T3>T1>NPK>T4>CK, and the T2 treatment was significantly higher than the other treatments, 71.35 cm^2^, 172.87 cm^2^, 208.56 cm^2^, 297.36 cm^2^, and 400.87 cm^2^ than the T3, T1, NPK, T4, and CK treatments, respectively. The sizes of root volume of the various treatments were expressed T2>T3>NPK>T1>T4>CK, T2 treatment was higher than the other treatments, in which the difference between NPK treatment and T1 treatment was not significant, and T2 was 23.32%, 46.03%, 54.16%, 97.07%, and 243.83% higher than the treatments of T3, NPK, T1, T4, and CK, respectively. The size of root tip number of each treatment showed that T2>T3>NPK>T1>T4>CK, and T2 treatment was higher than the other treatments, among which, there was no significant difference between NPK and T1 treatments, and the T2 treatment was 26.04%, 42.04%, and 54.53% higher than the T3, NPK, and T1 treatments, respectively. Root indexes corresponded to root morphology. Taken together, the root indexes and morphology of tobacco plants showed a tendency of first increasing and then decreasing with the increase of the application of potassium fulvic acid, and the application of appropriate dosage of potassium fulvic acid was conducive to the uptake of nutrients and water by the tobacco plant, and promoted the growth of tobacco plants.

##### 3.6.3.2 Effect of different fertiliser treatments on root vigour of tobacco plants

**Figure 9:**
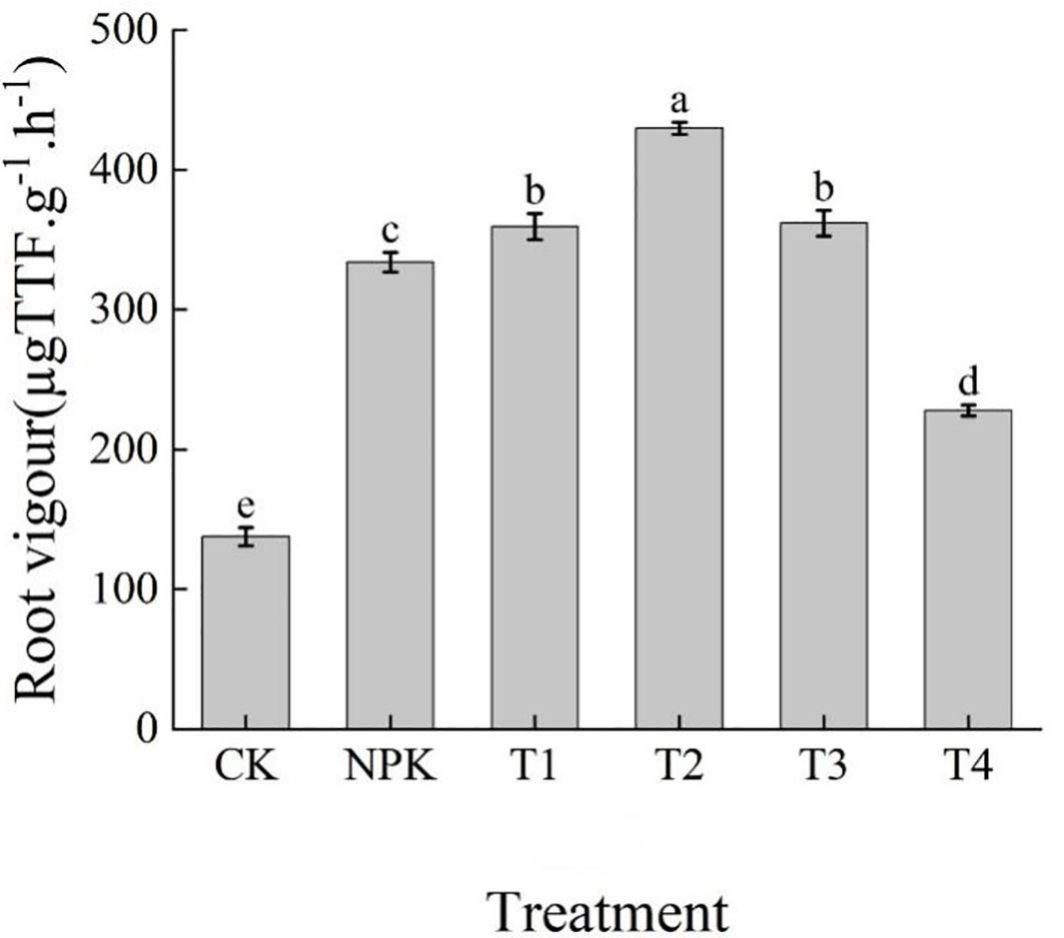
Effects of different fertilisation treatments on root activity of tobacco. Lowercase letters Represents an annotation of the significance level of the data.

As can be seen from **Figure 9**, the fertilisation treatments could improve the root vigour of tobacco plants to different degrees, and there were significant differences in root vigour among the treatments, with the size of root vigour of each treatment showing T2>T3>T1>NPK>T4>CK, and the T2 treatment was significantly higher than the other treatments, with an increase of 18.74%, 19.54%, 28.71% over T3, T1, NPK, T4, and CK, respectively, 88.58% and 211.87%, respectively (**Table 5**). The results indicated that the root vigour of tobacco plants showed a tendency of increasing and then decreasing with the increase of the application of potassium fulvic acid fertiliser, and that the application of appropriate dosage of potassium fulvic acid fertiliser could improve the root vigour of tobacco plants.

**Table 5:** Effect of different fertilisation treatments on photosynthetic characteristics of tobacco.

Time	Treatment	Pn (µmol.m^-2.^s^-1^)	Tr (mmol.m^-2.^s^-1^)	Gs (mmol.m^-2.^s^-1^)	Ci (µmol.mol^-1^)
Day 20 after transplanting	CK	17.7 ± 0.58d	5.98 ± 0.12d	305.67 ± 4.9c	205.97 ± 4.09c
	NPK	19.07 ± 0.47c	6.37 ± 0.14c	316.49 ± 5.71c	217.13 ± 5.51c
	T1	19.74 ± 0.64bc	7.02 ± 0.03b	334.82 ± 4.95b	222.13 ± 15.18c
	T2	21.76 ± 0.61a	8.04 ± 0.05a	388.65 ± 9.62a	280.63 ± 17.99a
	T3	20.17 ± 0.52b	7.05 ± 0.07b	336.23 ± 3.58b	248.97 ± 9.51b
	T4	18.11 ± 0.25d	6.10 ± 0.25d	315.46 ± 4.59c	209.87 ± 9.26c
Day 40 after transplanting	CK	18 ± 0.67d	6.06 ± 0.05d	308.33 ± 5.12d	208.63 ± 5.51d
	NPK	19.6 ± 0.57c	6.39 ± 0.42c	329.82 ± 10.05c	220.47 ± 13.62d
	T1	20.5 ± 0.57bc	7.07 ± 0.1b	349.82 ± 9.99b	240.8 ± 10c
	T2	23.2 ± 0.68a	8.08 ± 0.1a	412.65 ± 7.16a	300.63 ± 4.97a
	T3	21.16 ± 0.73b	7.13 ± 0.1b	352.89 ± 9.84b	260.47 ± 10.01b
	T4	18.41 ± 0.57d	6.20 ± 0.05c	320.46 ± 4.77d	215.53 ± 10.06d
Day 60 after transplanting	CK	19.2 ± 1.16d	6.2 ± 0.15d	321.33 ± 15.74d	217.3 ± 8.29d
	NPK	21.4 ± 0.83bc	6.83 ± 0.26c	356.49 ± 15.22bc	245.47 ± 10.02cd
	T1	22.4 ± 0.83b	7.57 ± 0.25b	379.82 ± 19.95b	279.13 ± 15.28c
	T2	24.8 ± 0.55a	8.35 ± 0.22a	474.32 ± 20.33a	318.97 ± 13.94a
	T3	22.5 ± 1.06b	7.66 ± 0.25b	376.23 ± 15.14b	292.53 ± 5.19b
	T4	20.21 ± 0.57cd	6.52 ± 0.14cd	338.13 ± 5.40cd	229.13 ± 10.06d

Lowercase letters Represents an annotation of the significance level of the data.

### 3.7 Effects of different fertilisation treatments on photosynthetic characteristics of tobacco plants

#### 3.7.1 Effects of different fertilisation treatments on net photosynthetic rate of tobacco plants

Photosynthetic indexes of tobacco plants were measured at different time periods, and the results of the experiment showed that at different time periods, all the fertilisation treatments significantly improved the photosynthetic indexes of tobacco plants compared with the control group. At the time of 60d, the treatments had the best photosynthetic indexes with an upward trend. Meanwhile, the T2 treatment, compared with conventional fertilisation, had the highest indicators of tobacco plants. The results indicated that the appropriate amount of potassium fulvic acid could effectively promote the growth of tobacco plants in the late stage, and too much or too little potassium fulvic acid could not promote the growth of tobacco plants well. Overall, the appropriate amount of potassium fulvic acid can effectively improve the growth and development of tobacco plants, increasing yields to ensure increased economic benefits.

#### 3.7.2 Effect of different fertilisation treatments on chlorophyll content of tobacco plants

**Figure 10:**
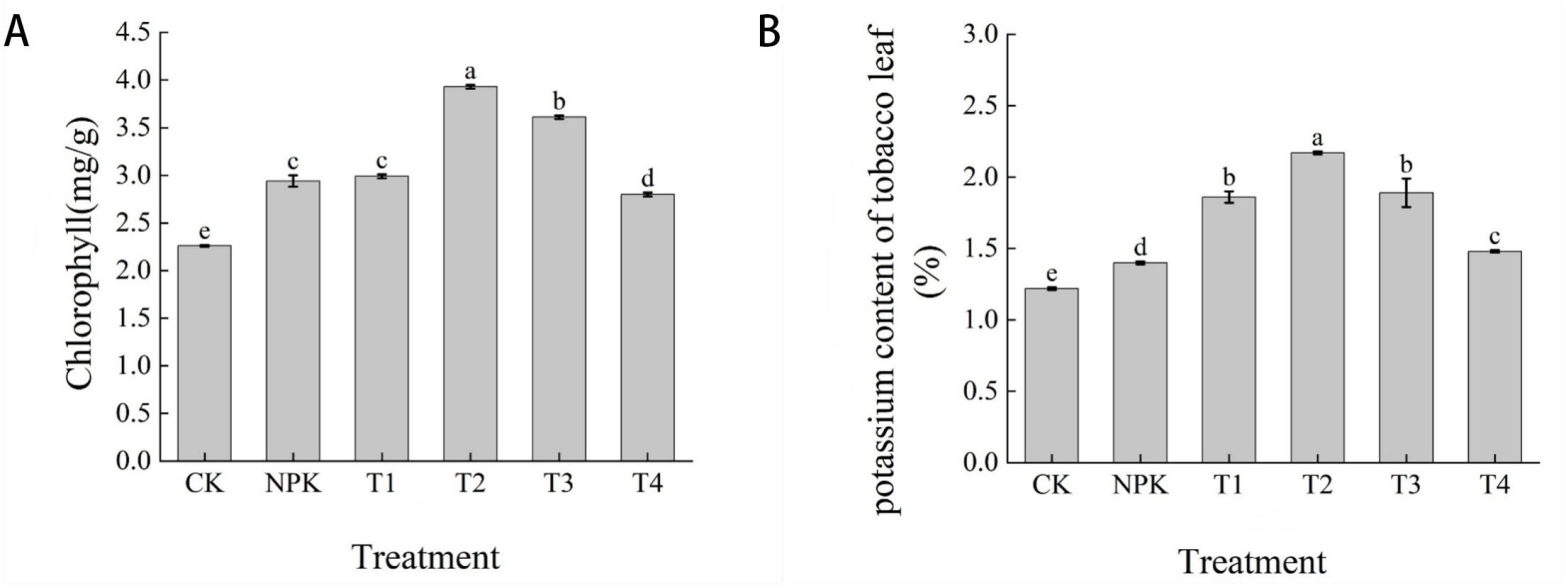
Effects of different fertilisation treatments on chlorophyll **(A)** and potassium **(B)** content in tobacco leaves. Lowercase letters Represents an annotation of the significance level of the data.

Chlorophyll content is closely related to photosynthesis, and the amount of chlorophyll affects the intensity, rate and efficiency of photosynthesis. Usually, the higher the chlorophyll content of the plant, the stronger its ability to photosynthesis. As shown in **Figure 10A**, the magnitude of chlorophyll content of each treatment showed that T2 > T3 > T1 > NPK > T4 > CK, and the difference between T2 and other treatments was significant, and the highest chlorophyll content was found in the T2 treatment, which was higher than the other treatments by 8.14%, 23.92%, 25.19%, 28.75% and 42.49%. The results indicated that the application of appropriate dosage of potassium fulvic acid could improve the photosynthetic capacity of leaves.

### 3.8 Effect of different fertiliser treatments on potassium content of tobacco leaves

As can be seen from **Figure 10B**, on the 65th day after transplanting, the size of the potassium content of tobacco leaves in each treatment showed that T2>T3>T1>T4>NPK>CK, T2 treatment was the highest, 2.17%, and CK treatment was the lowest, 1.22%, and the potassium content of tobacco leaves in T2 treatment was significantly higher than that of other treatments, in which the potassium content of tobacco leaves in CK and NPK treatments was lower than that of each potassium fulvic acid. The application of appropriate dosage of potassium fulvic acid could increase the potassium content of tobacco leaves.

### 3.9 Effect of different fertiliser treatments on tobacco plant diseases

As can be seen from **Table 6**, the incidence rate of tobacco virus disease and the size of the disease index were CK>NPK>T4>T1>T3>T2, in which the incidence rate and index of T2 treatment were the smallest, and the T2 treatment was good at preventing and controlling the tobacco virus disease, which was 62.50%, 54.72%, 52.04%, 33.33%, and 22.48% more than the other treatments, respectively; and the incidence rate and size of the disease index of the tobacco rootstock. The incidence rate and disease index of the disease were CK>NPK>T4>T3>T1>T2, in which the incidence rate and disease index of T2 treatment were the smallest, and the T2 treatment was effective in the prevention and control of tobacco rootstocks, which improved 55.26%, 46.85%, 45.14%, 41.37% and 31.97% compared with the other treatments, respectively. The results of the data indicated that T2 treatment was the most effective in the control of tobacco virus diseases and tobacco root and stem diseases, and the application of appropriate amount of potassium fulvic acid could reduce tobacco diseases.

**Table 6:** Effects of different fertilisation treatments on tobacco plant diseases.

Treat	Tobacco virus disease		Tobacco root disease	
	Morbidity rate (%)	Disease index (%)	Morbidity rate (%)	Disease index (%)
CK	83.33	53.33	50	31.67
NPK	76.67	44.17	43.33	26.66
T1	60	30	33.33	20.83
T2	43.33	20	23.33	14.17
T3	50	25.8	40	24.17
T4	70	41.7	36.67	25.83

## 4 Discussions

### 4.1 Potassium fulvic acid improves nutrients in mono-cropped soil and promotes tobacco plant growth

Tobacco continuous cropping experiment showed that with the increase of continuous cropping years, the soil organic matter content was basically unchanged, the pH decreased, the quick-acting nitrogen, phosphorus and potassium content generally showed a decreasing trend, and the content of most trace elements showed a decreasing trend. This may be related to the acidification of tobacco continuous cropping soil. Potash has been an important tool for improving crop performance and health for decades, but there is increasing concern about soil acidification, declining soil nutrients and declining microbial activity. Soil properties and nutrient levels directly affect plant growth and productivity (Ma et al., 2021). The beneficial effects of potassium fulvic acid in improving plant growth performance and soil improvement have been widely recognized (Abbey et al., 2018; Wang et al., 2020; Giovanardi et al., 2016; Pradip et al., 2016). In this study, the potassium fulvic acid treatment significantly improved the soil physicochemical properties, and the T2 treatment was significantly higher than the other treatments, increasing the effectiveness of soil nutrients for plants. Soil enzymes are the main driving factors of soil nutrient metabolism, which can directly indicate the degree and direction of soil nutrient transformation and biochemical processes (Aponte et al., 2020; Song et al., 2024). S_CAT is an important indicator of the soil microbiological environment, which is related to the intensity of soil respiration and soil microbial activity, and is effective in preventing the toxicity of hydrogen peroxide (Aponte et al., 2020; Shang et al., 2021). In this study, T2 treatment significantly enhanced S_CAT activity compared to CK and NPK treatments, which was supported by several studies (Zhu et al., 2021). In addition, S_UE promotes the hydrolysis of urea to ammonia, which provides nitrogen nutrition to plants (Li et al., 2022a). S_SC can catalyse the hydrolysis of soil oligosaccharides into plant-available monosaccharides such as glucose and fructose, and participate in the soil organic carbon cycle (Vuyyuru et al., 2019), T2 treatment significantly increased S_UE and S_SC activities compared to CK and NPK treatments, which may be due to the increased abundance of beneficial rhizosphere microorganisms. The results showed that T2 treatment had significant effects on soil S_CAT, S_UE, S_SC activities and soil physicochemical properties in this study. In conclusion, moderate amount of potassium fulvic acid significantly improved soil enzyme activities and soil physicochemical properties under continuous cropping soil.

The effects of tobacco continuous cropping barriers on agronomic traits of tobacco plants are the most intuitive. Long-term continuous cropping of tobacco will lead to the deterioration of tobacco agronomic traits, which seriously affects the quality of tobacco production and production value (Chen et al., 2024). After the application of suitable concentration of fulvic acid, by the action of its active groups, improve the continuous crop soil nutrients and soil enzyme activity, thus promoting the uptake of the plant, while increasing the photosynthesis in the leaves, enhancing the efficiency of photosynthesis, thus improving the yield and quality of crops, enhancing the resistance of the plant to pests and diseases, and strengthening the plant’s resistance to adversity. It can replace chemical fertiliser and does not pollute the environment (Zheng et al., 2021; Sun et al., 2022; Wang and Xiong, 2022). In this study, six treatments were established and the results of the experiment showed that in the CK treatment group, soil nutrients were decreased, agronomic traits, photosynthetic characteristics, root indexes, and chlorophyll of plants were decreased, growth plants of tobacco grass were inhibited, and potassium content of tobacco leaves was decreased. In T2 treatment groups, soil nutrients were increased, agronomic traits, photosynthetic characteristics, root indexes, chlorophyll, etc. were significantly higher, the growth of cigartobacco tobacco grass plants was better, and the potassium content of tobacco leaf was also increased, while all T3 treatments decreased relative to T2 treatments. The results showed that the agronomic traits, photosynthetic characteristics, root indexes, chlorophyll and soil nutrients of tobacco plants were positively correlated with the amount of potassium fulvic acid applied within a certain range, and the growth was inhibited after exceeding a certain range. Plant height, stem thickness, leaf length and leaf width are important agronomic traits of tobacco, which can reflect the growth and appearance changes of tobacco to a certain extent. Meanwhile, chlorophyll, as the main pigment for photosynthesis in plants, its content is one of the important factors determining the photosynthetic capacity of plants, and the photosynthetic rate can also reflect the ability of plants to carry out photosynthesis, and these indexes are important factors weighing the good or bad growth of tobacco. The biomass accumulated by plants through photosynthesis can be directly reflected in these indicators, which may be because the application of a certain amount of potassium fulvic acid can promote the development of the plant root system and the enhancement of the nutritional capacity, and increase the net photosynthetic rate of tomato, while more than this amount of these promotional effects are not obvious or inhibitory effect (Li et al., 2016). High concentration of potassium fulvic acid inhibits plant growth because crops in the seedling stage, the root system has just sprouted soon, the number of roots is small, the absorption area is small, the absorption capacity is weak, the high concentration of fertiliser will cause damage to the root system of the seedling, affecting the subsequent growth of the plant. From the analysis of the growth of tobacco plant by the potassium fulvic acid, the suitable concentration of potassium fulvic acid is significantly better than the conventional fertilisation to promote the growth of tobacco plant. Therefore, the test results showed that the T2 treatment was more effective in improving the continuous soil and more suitable for plant growth.

### 4.2 Effect of potassium fulvic acid on the rhizosphere soil microbial community of continuous cropping soils

Disruption of soil microbial community structure by soil succession is an important cause of the formation of succession barriers, and soil rhizosphere microbes play a key role in maintaining soil quality and influencing host plant growth (Shang et al., 2021; Zheng et al., 2021; Sun et al., 2022. fulvate fertiliser is a good soil conditioner, which can also be made to function as a regulator of the structural composition of microbial communities due to the multiple bioactive functions of fulvic acid. The application of potassium fulvic acid fertiliser significantly affected the overall diversity of bacterial and fungal communities in the rhizosphere soil of tobacco plants.

The abundance and diversity of soil microorganisms are quite important indicators of soil ecology. In the present study, T2 treatment reduced the bacterial Chao 1 index of the rhizosphere soil samples as compared to CK, indicating that potassium fulvic acid, affected the bacterial diversity. NMDS showed that potassium fulvic acid, significantly altered the composition of the rhizosphere microbial community in tobacco plants. The study showed that the effect of potassium humate and fulvic acid on bacterial and fungal communities (Jin et al., 2022). Thus, the effect of potassium fulvic acid, on the rhizosphere microbial community is favourable to ameliorate the effect of continuous cropping soils on tobacco plants.

In rhizosphere samples, at the phylum level, the dominant bacterial phylum was Proteobacteria more likely to survive in environments rich in multiple nutrients (Trivedi et al., 2013). Bacteria in actinomycetes enhance host nutrient acquisition and protect plants from various abiotic stresses (Ye et al., 2015). Actinomycetes have been found to be widespread in plants as well, and similarly, actinomycetes are decomposers of organic matter (Ferreira et al., 2017). Fertilisation treatments increased the relative abundance of Bacillus phylum, an rhizosphere beneficial bacterium, which plays an important role in soil improvement, plant growth promotion, disease resistance and enhancement of salt tolerance in plants (Liu et al., 2020; Shang et al., 2021). The dominant fungal phylum is Ascomycetes, which promotes the uptake of nutrients by the root system of the plant, thus promoting plant growth (Li et al., 2022b). We have discovered microorganisms that may be associated with disease, such as Fusarium and Potyphomonas. The prophylactic bacterium Mortierellomycota was also discovered. At the genus level, the T2 treatment decreased the relative abundance of *Fusarium* and the T3 treatment increased the abundance of *Fusarium* spp. This is in line with the investigated tobacco incidence and disease index suppression of the T3 treatment. In this study, it was found that the addition of potassium fulvic acid to the soil increased the abundance of beneficial bacteria in tobacco soil. This is supported by several studies (Jin et al., 2022). It can provide guidance and a molecular basis for refining the mechanism of soil nutrient regulation and disease prevention in the tobacco succession.

Changes in soil nutrient status can directly affect the morphological structure and function of the rhizosphere microbial community, and its feedback effect gradually affects the nutrient status and quality of the soil (Sun et al., 2022; Yin et al., 2022). Therefore, this study assessed the correlation between rhizosphere nutrient status and dominant microorganisms. These results suggest that the changes of microbial communities affected by potassium fulvic acids is closely related to changes in soil nutrient status (OM, AN, NN, S_UE, S_SC and S_NPT), which is supported by several studies (Zheng et al., 2021; Sun et al., 2022; Yin et al., 2022). Studies show beneficial effects of potassium fulvic acid on plant growth and soil improvement (Giovanardi et al., 2016; Pavlova et al., 2020; Zhang et al., 2023). Overall, we found that potassium fulvic acid has a positive effect on plant growth.

## 5 Conclusions

In the present study, we revealed the beneficial effects of potassium fulvic acid in promoting tobacco growth and ameliorating continuous cropping soil, and the results showed that the application of 4.65 g/kg of potassium fulvic acid effectively improved the soil physicochemical properties and enzyme activities, changed the composition of soil rhizosphere microbial communities, and led to the reduction of diseases and the promotion of the growth and development of tobacco plants. Therefore, 4.65 g/kg of potassium fulvic acid and origin maybe recommended to improve soil health parameters and tobacco crop growth characteristics.

## Data Availability

The original contributions presented in the study are included in the article/supplementary material. Further inquiries can be directed to the corresponding author.
